# Hospital Annual Meetings

**Published:** 1919-05-17

**Authors:** 


### HOSPITAL ANNUAL MEETINGS.

### Hospital for Diseases of the Heart.

THE Committee of Management, at the annual meeting, reported that the debt to the bank of £8,300 had been paid off out of the legacy received from the late Mrs. Scott. On the other hand, the Committee was faced with expenditure in order to provide larger consulting-room accommodation in the out-patients' department, and an appeal was being made to friends to raise a fund of about £8,000 for that purpose. The enlargement of the wards would have to be dealt with later. Over 200 in-patients and £19,614 out-patients were cared for last year. Income amounted to £6,227, and expenditure to £6,242, leaving a small deficit of £15.

### Hospital for Nervous Diseases.

LORD CECIL MANNERS presided over the annual Court of Governors of the West End Hospital for Nervous Diseases, Welbeck Street. The present accommodation is insufficient and unsuitable for modern requirements. Schemes entered upon for securing an up-to-date building have so far not proved successful, but the Committee of Management feels compelled to bring the matter to a head, and trusts to receive generous support so soon as a working plan can be promulgated. Patients admitted in 1918 numbered 450, or 123 children, ninety-seven women, 115 men, and 115 soldiers. Out-patients increased to 3,760, with 36,133 attendances. In the massage and electricity department over 30,000 cases were dealt with. Expenditure last year was £12,995, as compared with £10,441 in 1917, while total income amounted to £12,675. There are now 102 beds at the hospital. The stay of civilian patients averaged seventy-five days, and those of soldiers 103.

### E. London Hospital for Children.

COLONEL CHARLES NEEDHAM presided over the annual Court of Governors, when it was reported that two events had marked the past year as the most memorable in the records of the institution. The first was the kindly interest of one who had augmented the funds by the most magnificent bequest ever received, and the second was the opening of an office in the West End with the object of making the hospital more widely known. The aim of that Appeal Office was to obtain funds for improvements in various departments, and for extending the building to meet the ever-increasing requirements of the East-End population. It was estimated that a sum of at least £50,000 would be required for that purpose. In-patients decreased in number last year, while new out-patient cases were more. Receipts from all sources exceeded those of the preceding year by nearly £4,000.

